# Comparison of outcomes and analysis of risk factors for non-union in locked plating of closed periprosthetic and non-periprosthetic distal femoral fractures in a retrospective cohort study

**DOI:** 10.1186/s13018-019-1204-z

**Published:** 2019-05-24

**Authors:** James Karam, Paul Campbell, Michael David, Michael Hunter

**Affiliations:** 1 Gosford District Hospital, Central Coast Local Health District, Gosford, NSW 2250 Australia; 2 School of Medicine and Public Health, University of Newcastle, Callaghan, NSW 2308 Australia

**Keywords:** Distal femur fracture, Periprosthetic fracture, Total knee arthroplasty, Locking plate fixation, Non-union, Comminution

## Abstract

**Background:**

The primary aim was to compare the outcomes of locked plating of closed distal femur periprosthetic and non-periprosthetic fractures by testing the hypothesis that outcomes would be worse in the periprosthetic group. The secondary aim of this study was to identify risk factors for non-union.

**Methods:**

A single-center study over an 8-year period utilizing a retrospective cohort design was performed. Sixty-eight patients with periprosthetic fractures and 57 patients with non-periprosthetic fractures met inclusion criteria for the study. There was a significant difference between groups in mean age (80.1 years periprosthetic vs. 70.9 years non-periprosthetic (*p* < 0.001)). Statistical analysis between groups was used to assess the outcomes of time to union, incidence of non-union, post-operative functionality, incidence of complications, progression to revision surgery, and mortality. A secondary multivariable analysis was used to assess risk factors for non-union and factors positively associated with union.

**Results:**

There were no significant differences in outcomes between groups. Union rates were 83.8% (57/68) in the periprosthetic group and 78.9% (45/57) in the non-periprosthetic group (*p* = 0.648). Comminution was identified as a significant risk factor for non-union (*p* = 0.005). Use of a submuscular technique had a significant positive association with union (*p* = 0.006).

**Conclusions:**

Outcomes of surgical treatment for periprosthetic and non-periprosthetic distal femur fractures are similar. There is a significant risk of non-union in locked plating of both groups.

## Background

Distal femur periprosthetic fractures have become a more common complication in association with increased rates of knee arthroplasty in an aging population [1]. The incidence of periprosthetic fracture is reported as 0.3–2.5% [2]. The rate of total knee arthroplasty in Australia as recorded on a national registry has increased 139.8% since 2003 [3]. The majority of these fractures are treated operatively, with locked plating a commonly used modality [4]. Other treatment options include retrograde intramedullary nailing and distal femoral arthroplasty as a primary treatment. There is no clear consensus on the optimal surgical intervention [5].

The aims of surgical treatment are to achieve stable anatomical reduction allowing for analgesia, ease of care, and early mobilization [6, 7]. These fractures have been shown to have problematic healing, with reported non-union rates of 6–25% [8, 9]. Other known problems associated with these fractures include decreased mobility and loss of independence in more than 50% of patients, a 3–37% risk of post-operative complication, and an up to 25% rate of 1-year mortality [10–13].

In most instances, periprosthetic fractures are treated similarly to their non-periprosthetic counterparts, where this fracture is common in the elderly osteoporotic population. To date, there has been little in the way of direct comparison of outcomes of both groups [14].

The hospital for this study, located in an area with a large retired population, has treated a large number of these fractures. The primary aim of this study was to compare the outcomes of surgical treatment, specifically locked plating, of periprosthetic and non-periprosthetic fractures. The secondary aim was to determine predictors of non-union and union in both groups.

It was hypothesized that overall outcomes in locked plating of periprosthetic fractures would be worse than their non-periprosthetic counterparts. Based on 2017 Australian registry data recording an average age at primary knee arthroplasty of 68.7 years and 92.7% of patients classified as American Society of Anesthesiologists (ASA) score 2 or 3 [3], it was hypothesized that patients with periprosthetic fractures may represent an older and more frail population than a non-periprosthetic fracture population. The overall value of this study will add to existing knowledge about this fracture and help aide surgeons in determining a common approach to both fracture groups.

## Methods

Local health district ethics approval was attained for the purpose of performing a retrospective cohort analysis of patients with distal femoral fractures treated in a single hospital from 2011 to 2018. A search was performed through the hospital medical records department using diagnosis-related group (DRG) codes for distal femoral and related fractures [15].

**Fig. 1:**
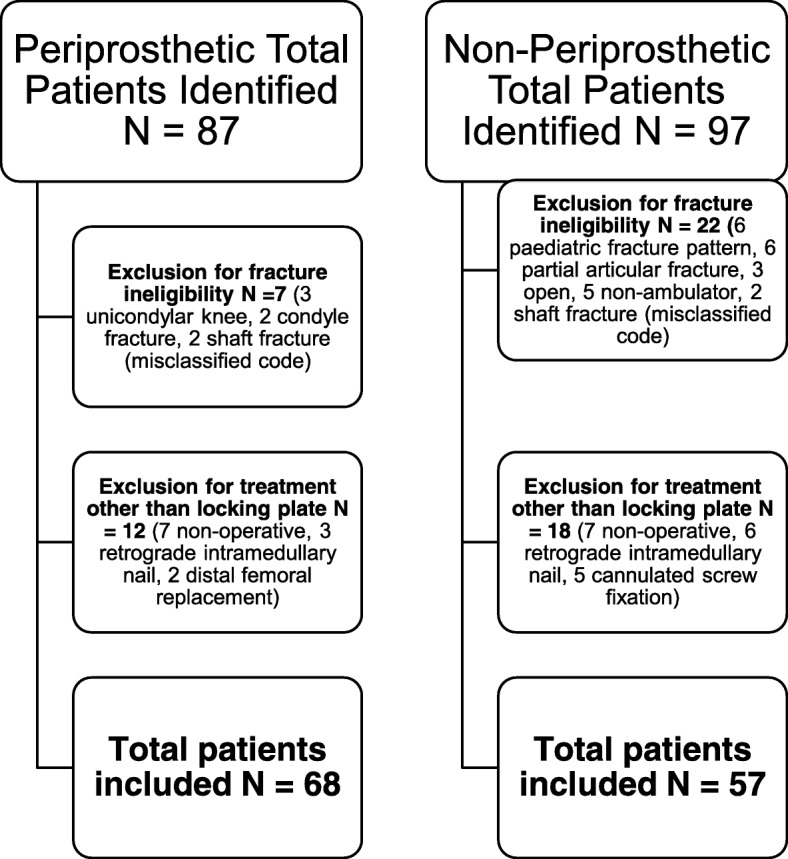
Patient selection

A flowchart of patients identified as eligible for the study can be found in Fig. 1. All patients aged 16 and above with either extra or intra-articular distal femoral fractures around either a prosthetic or non-prosthetic knee treated with locked plating only were included. Open fractures, pediatric fracture patterns, partial articular fractures (including Hoffa fractures, isolated condyle fractures, and intracondylar splits), fractures around a unicompartmental knee replacement, and fractures in non-ambulant patients were excluded. Patients who were treated non-operatively or with another modality (including retrograde intramedullary nail and distal femoral replacement) were also excluded.

All patients received anteroposterior (AP) and lateral x-rays at presentation to the emergency department and at follow-up. Computed tomography scans were obtained in the majority of patients to aid pre-operative planning. All fractures were sustained following low-energy falls. Fractures were classified according to the Arbeitsgemeinschaft für Osteosynthesefragen/Orthopedic Trauma Association (AO/OTA) system for distal femur fractures [16]. Fracture comminution was defined as the presence of at least one separate metaphyseal or diaphyseal cortical bone fracture fragment (AO/OTA classification 33A2 or 3 and 33C2 or 3).

Surgery was performed either by consultant surgeons or trainees under consultant supervision. All patients received locking plate fixation using the stainless steel condylar locking compression plate (LCP) 4.5/5.0 (DePuy Synthes, Oberdorf, Switzerland). Surgery performed with exposure of the fracture was recorded as ‘open’ and surgery using a minimally invasive plate osteosynthesis (MIPO) technique was recorded as ‘submuscular’ [17]. Plate length and working length in plate holes was recorded. Working length was defined as the distance in plate holes between the proximal and distal most screws on each side of the fracture. All patients received routine pre- and postoperative antibiotics and anticoagulation and were made non-weightbearing for a minimum of 6 weeks postoperatively.

Follow up took place at 2 weeks, 6 weeks, 3 months, and 6 months. Union was defined as the presence of a minimum of three out of four bridging cortices on AP and lateral x-rays at 6 month follow up [18]. Patients who missed their 6-month follow up yet went on to unite had ‘time to union’ recorded at the time of their last follow up. X-rays failing to meet the minimum requirement of bridging cortices at 6 months were recorded as non-unions. These patients had extended follow up with further x-rays confirming the presence of non-union.

Complications recorded included hardware failure, wound or periprosthetic infection, peri-implant fracture, symptomatic hardware prominence, and the incidence of deep vein thrombosis in the post-operative period. Post-operative function was determined based on mobility and dependence status at last follow up or most recent hospital visit. Decreased function was recorded if the patient had become dependent on a walking aid or if they could no longer live independently (including placement in an aged care facility). Mortality was recorded through the scrutinization of patient records on an electronic record system.

Descriptive statistics recorded included means and standard deviations for continuous variables and counts (percentages) for categorical variables. Statistical analysis of outcomes between groups was performed using Pearson Chi-square tests comparing the incidence of non-union, post-operative functionality, incidence of complications, progression to revision surgery, and mortality. A survival analysis using competing risk modeling was applied to the comparative analysis of time to union. An analysis of risk factors for non-union and factors positively associated with union was performed using a Firth stepwise logistic regression model with an inclusion threshold of 0.2. The assumption of proportional hazards and appropriate model specified were assessed using the Hosmer-Lemeshow test and the Pregibon link test respectively. All tests were two-sided and had a 0.05 level of significance. Statistical analysis was performed using STATA Version 15 software (StataCorp LLC, College Station, Texas United States of America).

## Results

### Baseline characteristics

The final number of patients after exclusions was 68 in the periprosthetic group and 57 in the non-periprosthetic group. All patients were followed up for a minimum of 3 months (range 3–89 months, 22 months mean follow up), including patients who died within 3 months of their operation. Nine patients in the periprosthetic group and six patients in the non-periprosthetic group were considered as lost to follow up for the purpose of recording fracture union as these patients attended follow up in a different location and x-rays were not accessible. These patients were included in the overall analysis as details regarding other outcomes such as postoperative mobility and mortality were accessible via an electronic record system for later unrelated attendances at the study hospital location.

A summary of patient baseline characteristics can be found in Table 1. In the periprosthetic group, mean age was 80.1 years (standard deviation (SD) 9.53; 95% confidence interval (CI) 77.9–82.2) with 57 females and 11 males. In the non-periprosthetic group, mean age was 70.9 years (SD 18.6; 95% CI 66.4–75.3) with 44 females and 13 males. *T* tests for continuous variables and Fisher’s exact test for categorical variables were applied to detect significant differences in baseline characteristics between groups, finding a significant difference in age (*p* < 0.001) between groups and a significantly greater number of patients with alcohol dependency in the non-periprosthetic group (*p* = 0.008).

**Table 1:** Patient baseline characteristics

Baseline characteristic	Periprosthetic *N* = 68	Non-periprosthetic *N* = 57	*p* value
Average age (years)	80.1	70.9	< 0.001
Gender	57 Female (83.8%)	44 Female (77.2%)	0.530
Pre-operative walking aid requirement/dependant living arrangement	36 (53.0%)	28 (49.1%)	0.407
Average BMI	27.8	25.8	0.165
Smoking	16 (23.5%)	15 (26.3%)	0.610
Alcohol dependence	6 (8.8%)	15 (26.3%)	0.008
Diabetes mellitus	17 (25.0%)	9 (15.8%)	0.146
Steroids	9 (13.2%)	4 (7.0%)	0.165

*p* values calculated using *T* test and Fisher’s exact test

Fracture comminution was present in 36/68 (53.0%) periprosthetic fractures and 24/57 (42.1%) non-periprosthetic fractures. Further, 32/68 (47.0%) patients in the periprosthetic group and 29/57 (51.0%) patients in the non-periprosthetic group were treated using a submuscular or MIPO technique. Average plate and working length in the periprosthetic group was 12.6 and 4.6 holes respectively and 11.7 and 4.2 holes in the non-periprosthetic group.

### Comparison of outcomes of locked plating of periprosthetic and non-periprosthetic fractures

A summary of main outcomes recorded for both groups can be found in Table 2. Mean time to union in the periprosthetic group was 4.2 months (SD 1.2 months) and 4.7 months (SD 1.5 months) in the non-periprosthetic group. A survival analysis using competing risk modeling showed no significant difference in time to union (*p* = 0.827; 95% confidence interval: 0.70–1.55). Fifty-seven patients (57/68, 83.8%) in the periprosthetic group and 46 patients (46/57, 80.7%) in the non-periprosthetic group achieved union, with 11 and 12 patients respectively in each group going on to non-union. Conversely, the non-union rate was 16.2% in the periprosthetic group and 19.3% in the non-periprosthetic group. A Pearson chi-square test found no significant difference (*p* = 0.648) between groups with respect to non-union.

**Table 2:** Outcomes

Outcome	Periprosthetic *N* = 68	Non-periprosthetic *N* = 57	*p* value
Mean time to union (months)	4.2 (SD 1.2)	4.7 (SD 1.5)	0.827
Non-union	11 (16.2%)	12 (21%)	0.648
Complication	10 (14.7%)	9 (15.8%)	0.944
Revision	9 (13.2%)	8 (14.0%)	0.948
Post-operative worse mobility/dependant living arrangement	30 (44.1%)	23 (40.4%)	0.800
Mortality	15 (22.1%)	15 (26.3%)	0.566

*p* values calculated using survival analysis for mean time to union and Chi-squared test for remaining outcomes

A high proportion of patients (periprosthetic 30/68, 44.1%; non-periprosthetic 23/57, 40.4%) had decreased function at long-term follow up. Fifteen patients in each group (periprosthetic 15/68, 22.1%; non-periprosthetic 15/57, 26.3%) had died by the time the study was conducted. Chi-squared tests between both groups with respect to the incidence of decreased functionality (*p* = 0.800) and mortality (*p* = 0.566) showed no significant differences.

Ten patients (10/68, 14.7%) in the periprosthetic group and nine patients (9/57, 15.8%) in the non-periprosthetic group had recorded complications. A summary of recorded complications can be found in Table 3. The most common complication was plate failure and this was associated with non-union in 4/5 patients in the periprosthetic group and 2/4 patients in the non-periprosthetic group. Fisher’s exact test (*p* = 0.944) showed no significant differences between groups with regards to incidence of complication.

**Table 3:** Complications

Complication	Periprosthetic *N* = 10	Non-periprosthetic *N* = 9
Plate failure	5	4
Infection	1	2
Deep vein thrombosis	2	0
Symptomatic hardware prominence requiring removal	0	2
Peri-implant fracture	0	1
Pseudoaneurysm formation	2	0

A similar number of patients in both groups (periprosthetic 9/68, 13.2%; non-periprosthetic 8/57, 14.0%) required revision surgery. A summary of revisions can be found in Tables 4 and 5. The commonest indication for revision was non-union. Fisher’s exact test (*p* = 0.948) showed no significant differences between groups with regards to progression to revision surgery.

**Table 4:** Periprosthetic revisions

Revision	Indication	Periprosthetic *N* = 9
Revision fixation with bone graft	Non-union	4
Distal femoral replacement	Non-union	4
Revision total knee replacement	Tibial component loosening	1

**Table 5:** Non-periprosthetic revisions

Revision	Indication	Non-periprosthetic *N* = 8
Revision fixation with bone graft	Non-union	2
Revision fixation with bone graft	Peri-implant fracture	1
Arthrodesis nail	Infected non-union	1
Distal femoral replacement	Non-union	1
Total knee replacement	Progression of osteoarthritis	3

### Analysis of risk factors for non-union/positive association with union

A summary of information regarding baseline characteristics, fracture types, and operative details for patients who went on to non-union can be found in Tables 6 and 7. Risk factors for non-union assessed included age, gender, body mass index (BMI), smoking, diabetes mellitus, steroid medication use, alcohol dependence, fracture comminution, short plate, and working length (set at ten and four holes respectively) and use of an open technique.

**Table 6:** Periprosthetic non-unions

Patient	Age	Gender	Fracture classification	BMI	Smoking	Diabetes mellitus	Steroid medication	Alcohol dependence	Plate length (holes)	Working length (holes)	Open (i.e., ORIF)/submuscular plate insertion (i.e., MIPO)	Revision
1	81	F	33A3	29.8	Y	N	Y	Y	6	1	ORIF	Retrograde nail + graft
2	73	F	33A2	24	N	N	N	Y	16	2	ORIF	Distal femoral replacement
3	89	F	33A1	N/A	N	Y	N	N	10	4	ORIF	No
4	92	F	33A1	32	N	N	Y	N	16	6	MIPO	No
5	92	F	33A2	33.2	N	Y	N	N	16	4	MIPO	Retrograde nail + graft
6	90	F	33A3	24.2	Y	N	N	N	14	8	MIPO	No
7	85	M	33A3	20	N	N	N	N	12	4	ORIF	Distal femoral replacement
8	61	M	33A3	48	N	Y	N	N	12	6	ORIF	Revision plate + graft
9	81	F	33A3	30	N	Y	N	N	14	2	ORIF	No
10	79	F	33A3	22.1	Y	N	Y	N	12	5	ORIF	Distal femoral replacement
11	75	F	33A3	N/A	N	Y	Y	N	14	4	ORIF	No
Average	81.6	81.8% female	81.8% comminution	29.3	27.3% smoking	45.5% diabetic	36.4% steroid medication use	18.2% alcohol dependence	12.9	4.2	72.7% ORIF

**Table 7:** Non-periprosthetic non-unions

Patient	Age	Gender	Fracture classification	BMI	Smoking	Diabetes mellitus	Steroid medication	Alcohol dependence	Plate length (holes)	Working length (holes)	Open (i.e., ORIF)/submuscular plate insertion (i.e., MIPO)	Revision
1	51	F	33A2	N/A	N	N	N	N	10	5	ORIF	No
2	95	F	33A1	22	N	N	N	N	12	6	MIPO	No
3	93	F	33C2	N/A	N	N	N	N	10	4	ORIF	No
4	56	F	33C1	N/A	Y	N	N	Y	8	3	ORIF	No
5	48	M	33A1	N/A	Y	N	N	Y	6	2	ORIF	Arthrodesis
6	91	F	33C2	16	Y	N	N	N	6	2	ORIF	No
7	47	F	33A3	40	Y	Y	N	N	12	3	ORIF	Revision plate + graft
8	87	F	33A3	22.5	N	N	N	N	12	4	MIPO	No
9	78	F	33A3	35	N	N	N	N	12	5	MIPO	No
10	60	F	33A3	24.5	N	N	N	N	14	7	ORIF	Distal femoral replacement
11	43	M	33A3	23	Y	N	N	Y	14	3	ORIF	Retrograde nail + graft
12	86	F	33C2	21.9	N	N	N	N	14	3	ORIF	No
Average	69.6	83.3% female	75.0% comminution	25.6	41.7% smoking	8.3% diabetic	0.0% steroid medication	25.0% alcohol dependence	10.8	3.9	75.0% ORIF	

A summary of the statistical analysis of risk factors for non-union and positive associations with union can be found in Tables 8 and 9. A Firth multivariable logistic model identified only comminution as a significant risk factor for non-union (*p* = 0.005). Other risk factors were found to be non-significant. Patients with comminuted fractures had a more than four times greater risk of non-union compared to patients with non-comminuted fractures (odds ratio 4.60; 95% confidence interval 1.60–13.17). The same multivariable model was applied to the above factors looking for positive association with union. Use of a submuscular technique (*p* = 0.006) and non-comminuted fracture patterns (*p* = 0.014) were significantly associated with achievement of union. A positive association with union was observed with use of a plate equal to or longer than ten holes in length (*p* = 0.065); however, this result was not statistically significant. Younger age, BMI (equal to or less than 25), gender, absence of smoking/diabetes/steroid use/alcohol dependence, and longer plate working length (equal to four holes or greater) were not found to have a significant positive association with union.

**Table 8:** Statistical analysis of risk factors for non-union

Risk factors for non-union	*p* value
Male sex	0.424
Age ≥ 75	0.421
BMI > 25	0.235
Smoking	0.237
Diabetes mellitus	0.494
Steroid medication	0.138
Alcohol dependence	0.829
Fracture comminution	0.005
Open procedure	0.150
Plate length < 10	0.529
Working length > 4	0.227

*p* values calculated using backwards stepwise logistic regression multivariable analysis

**Table 9:** Statistical analysis of positive associations with union

Positive association with union	*p* value
Male sex	0.343
Age < 75	0.743
BMI ≤ 25	–^a^
Non-smoker	–
Nil diabetes mellitus	–
Nil steroid medication	–
Nil alcohol dependence	–
Simple fracture pattern	0.019
MIPO procedure	0.008
Plate length ≥ 10	0.065
Working length ≥ 4	–

^a^Variables without listed *p* values were not found to be significant in the model building process and thus were not included in the final multivariable analysis

## Discussion

To date, this study is the largest direct comparison of the outcomes of surgical treatment of distal femoral periprosthetic and non-periprosthetic fractures. No significant differences were found in outcomes between groups. Only one other paper was found directly comparing the outcomes of locked plating in these two groups (Song 2013 [14]). Despite reporting overall better union rates, outcomes between groups were also similar.

Distal femoral fractures are a challenging group of injuries for the orthopedic surgeon to treat [19]. This study has reflected what is already known in the literature regarding high rates of non-union, decreased post-operative functioning, and increased mortality. In particular, an overall non-union rate in both groups of 17.8% was comparable to reported rates in previous studies.

It could not be shown that the outcomes in the periprosthetic group would be worse than the non-periprosthetic group. Despite a 10-year age difference between groups, an almost equal number of patients in each group (accounting for 51.1% of patients in total) were considered frail through a surrogate measure of pre-morbid dependence on walking aids or living in an aged care facility. This reflects the incidence of distal femoral fractures as part of a spectrum of fragility fractures. As part of this spectrum, many similarities can be drawn between distal femoral and proximal femoral or hip fractures. Unlike hip fractures however, there is no widely accepted treatment algorithm or standard of care [20]. With increasing rates of arthroplasty especially in younger patients, periprosthetic fractures can be expected to rise in incidence [21]. Based on equal outcomes reported between groups in this study, a common approach to both periprosthetic and non-periprosthetic fractures should be utilized in future treatment algorithms. A notable exception to this is in the choice of fixation with a retrograde intramedullary nail, where a closed box design in prosthetic knees may preclude use of such a device [22].

In the secondary analysis of risk factors for non-union, comminution was identified as a strong predictor of non-union. A systematic review of distal femoral non-unions showed metaphyseal comminution to be the most commonly associated fracture pattern [23]. The corollary to this observation—that simple fracture patterns are significantly more likely to progress to union, was also observed in the study. A positive association with union from use of a MIPO technique was demonstrated. This supports the theoretical benefit of submuscular plate insertion in preserving fracture site biology and therefore promoting better bone healing [24]. This relationship has been observed in a number of studies [25–27].

A positive trend toward union was observed in association with use of longer plates. This trend was not observed in conjunction with longer plate working lengths, where union rates are predicted to be improved as a result of reduced fixation rigidity, allowing for fracture site micromotion [28]. It is generally recommended that three to four holes should be left empty at the site of the fracture [29]. Although the average plate working length in both groups in this study was 4.4 holes, a positive relationship with union from this was not found. This is in keeping with a number of studies showing no or minimal correlation between longer working length and union [25, 30, 31]. Some studies have shown better healing with titanium versus stainless plates, proposed to be associated with reduced material stiffness [32, 33]. This relationship was not examined in this study as stainless steel plates were exclusively used.

**Fig. 2:**
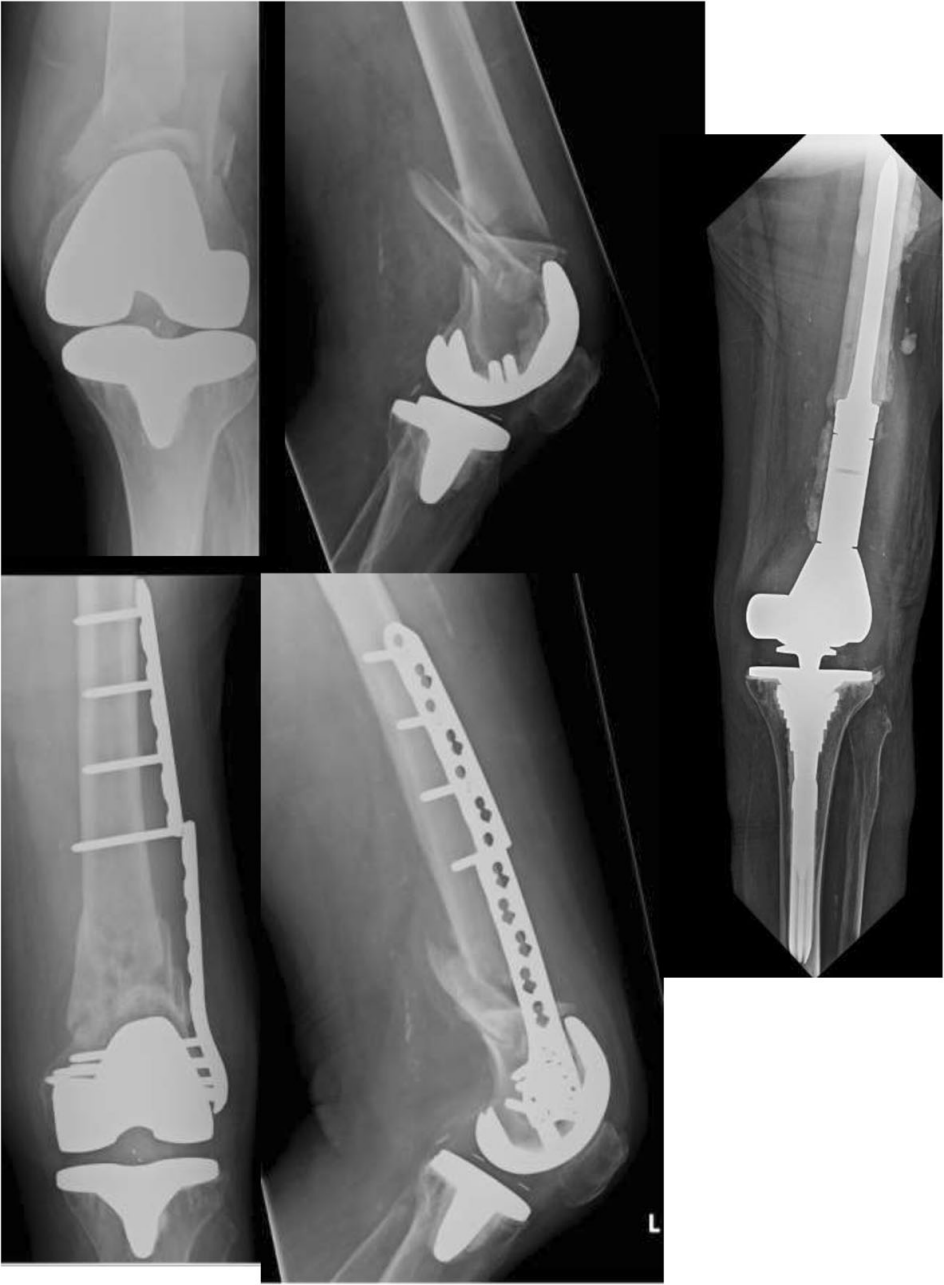
Case example. It this case, the patient received a locking plate via an open technique with indirect reduction. The patient proceeded to non-union at 6-month follow up with consequent plate failure and was revised to distal femoral replacement

Locked plating as applied to distal femoral fractures is still an evolving technique [34]. The benefit of locking plate technology in creating a fixed angle construct is attractive, especially as applied to elderly osteoporotic fracture patterns. In practice, plates are often applied without direct bony contact, in principle an ‘internal’ external fixator’ [35]. This is often a necessity in periprosthetic fractures where the position of the femoral component may preclude optimal plate positioning. Although a number of studies have shown difficulties in achieving optimal plate fit in locking plate designs, there is as yet no clinical evidence to show any downside to this [7, 36]. As demonstrated in this study however, without bony union, locking plates still have a tendency to fail. There appears to be a beneficial effect on union from use of a submuscular technique, and use of this technique should be encouraged whenever possible, especially in simple fracture patterns. Achieving anatomical reduction in highly comminuted fractures may not always be achievable with lateral locking plates applied with a strictly MIPO technique. In particular, there exists concern for fixation failure and varus collapse in patients with medial comminution [37]. A case example in Fig. 2 is illustrative. In this setting, there may be a role for intramedullary fibular strut allograft use and or addition of a medial plate [38, 39]. Various aids to reduction in using MIPO technique, such as joystick pins, percutaneous clamps, and maximizing use of the plate as a reduction tool, have been described [19]. Further work is needed to determine an acceptable degree of reduction in application of MIPO technique, and whether the benefits of achieving anatomical reduction outweigh the invasiveness of the approach.

An alternative to fixation is arthroplasty. The results in this study lend support to the rationale to perform distal femoral replacement for elderly patients with highly comminuted distal femoral fractures. The greatest benefit here is the ability to allow the patient to commence full weightbearing postoperatively. Furthermore, by virtue of the treatment, the problem of non-union is eliminated. Significant concerns for regular adoption of this practice include the safety of performing complex surgery on frail patients, an increased risk of associated complications most especially infection and costs involved [40–42]. Recent studies reporting on the viability of distal femoral replacement as an option are promising [43–45], and future research efforts are required to better prove the safety and efficacy of this practice.

The influence of post-operative weightbearing instructions on patient outcomes and union rates merits consideration. As the practice of the study location was to make all patients non-weightbearing for a minimum of 6 weeks, analysis was not possible in this regard. Although non-weightbearing is commonly the practice followed in many centres, recent studies [7, 46] have shown successful outcomes including high union rates in patients allowed full weightbearing after locked plating of distal femoral fractures. Although no biomechanical proof currently exists, there may be a beneficial effect on fracture site biology from weightbearing. There may be reluctance from surgeons to follow this practice, especially as locking plates are by definition load-bearing and not load-sharing devices and thus there may be concerns for early plate failure. However, there is much to commend the practice of allowing full weight-bearing after treatment of distal femoral fractures, especially in elderly patients where avoidance of prolonged bed rest and its associated complications is desirable. This should be a standard of care in management of the elderly and future research efforts are needed to increase the clinical weight of evidence for this practice.

This study was limited by its retrospective design. Although locked plating was the predominant mode of fixation in the cohort of identified patients, exclusion of patients who were treated with different modalities may have created a selection bias. This study excluded patients with higher energy or open injuries representing the mostly younger end of the bimodal age distribution of distal femoral fractures. It was the intention of the study to focus on an elderly cohort with low energy closed injuries, and the results of this study should be applied specifically to this cohort. Despite overall consistency in locked plating operative technique at the study location, variations in technique between surgeons introduced heterogeneity to the reported results. Patient-reported outcomes were not performed, and ideally these should have been performed to determine the clinical significance of the results.

## Conclusions

In conclusion, no differences were found in outcomes of locked plating of periprosthetic and non-periprosthetic distal femoral fractures. Therefore, a common approach to both fractures is advocated. This will aid future research efforts in determining an acceptable algorithm for treatment of this fracture. Non-union remains the greatest challenge facing surgeons in management of distal femoral fractures. A significantly increased risk of non-union exists in patients with comminuted fractures. Surgeons should be aware of the potential for non-union in managing such patients. Future studies are needed to investigate efforts to improve union rates in locking plate fixation of highly comminuted fractures.

## Abbreviations

AO: Arbeitsgemeinschaft für Osteosynthesefragen
AP: Anteroposterior
ASA: American Society of Anesthesiologists
BMI: Body mass index
CI: Confidence interval
DRG: Diagnosis-related group
LCP: Locking compression plate
MIPO: Minimally invasive plate osteosynthesis
SD: Standard deviation
